# Delayed orthodontic care and its association with depression among Chinese adolescents: a cross-sectional study

**DOI:** 10.3389/fpsyt.2025.1541160

**Published:** 2025-05-02

**Authors:** Yang Li, Mengshan Yang, Botang Guo, Lusi Yang, Cui Xiaotian, Jiaxing Zhou, Kun Wang, Shouli Wang, Ping Tang, Shitao Dong

**Affiliations:** ^1^ Department of Stomatology, The Chengde Stomatological Hospital, Hebei, Chengde, China; ^2^ School of Economics, Zhejiang Gongshang University, Zhejiang, Hangzhou, China; ^3^ Department of General Practice, Shenzhen Luohu People’s Hospital (Luohu Clinical College of Shantou University Medical College), Guangdong, Shenzhen, China; ^4^ Department of Education, Qingdao Municipal Hospital, Qingdao, Shandong, China; ^5^ Department of Stomatology, The Xianxian Hospital of Traditional Chinese Medicine (TCM), Hebei, Cangzhou, China

**Keywords:** adolescents, orthodontic treatment, depression, mental health, treatment interventions

## Abstract

**Objective:**

Adolescents undergoing orthodontic treatment often face psychological challenges, and delays in treatment may exacerbate these issues. This study explores the prevalence of depressive symptoms among Chinese adolescents receiving orthodontic care and examines the potential link between treatment delays and depressive symptoms.

**Methods:**

A cross-sectional study was conducted among adolescent orthodontic patients in Chengde, Hebei Province. Data on demographic factors, treatment delays, and depressive symptoms were collected through structured questionnaires. The Beck Depression Inventory for Youth (BDI-Y) was used to assess depressive symptoms. Logistic regression analysis was performed to evaluate the association between delayed treatment and the risk of depressive symptoms.

**Results:**

The study found that 32% of adolescent orthodontic patients exhibited depressive symptoms, a rate notably higher than the general adolescent population. Furthermore, 82% of the patients experienced treatment delays. Factors such as age, gender, family income, parental marital status, treatment type, and malocclusion severity (IOTN score) were significantly associated with depressive symptoms. Logistic regression showed that delays of 181–360 days (*OR*=4.26, 95%CI: 1.37–13.28) and over 360 days (*OR*=3.40, 95%CI: 1.02–11.37) were significantly associated with a higher risk of depressive symptoms.

**Conclusion:**

Adolescents undergoing orthodontic treatment are at a heightened risk of developing depressive symptoms, particularly when facing significant treatment delays. Early intervention, including psychological support, education on treatment importance, and financial assistance, is essential to mitigate these risks and improve both oral and mental health outcomes.

## 1 Introduction

Adolescent malocclusion is one of the most common oral health issues worldwide, significantly impacting oral function, facial aesthetics, and overall health. Studies have shown that the global prevalence of malocclusion is particularly high among adolescents. A systematic review revealed that approximately 45-75% of adolescents globally suffer from varying degrees of malocclusion (1). In China, research indicates that the prevalence of malocclusion among 12-year-olds is as high as 67.8%, with 13.1% classified as severe cases (2). Malocclusion not only affects chewing function but can also lead to excessive tooth wear, periodontal disease, and an increased risk of dental caries (3). Moreover, the impact on facial aesthetics is especially pronounced, which may lower self-esteem and even lead to psychological problems in adolescents.

Orthodontic treatment is the primary method for correcting malocclusion, effectively restoring proper tooth alignment and bite function while improving facial aesthetics and enhancing the patient’s quality of life. Studies have demonstrated that adolescents who undergo orthodontic treatment experience improvements not only in oral function but also in mental health and social adaptation (4). However, despite the significant benefits of orthodontic treatment, delayed orthodontic treatment seeking remains common among adolescents.

Delayed orthodontic treatment refers to the postponement of treatment after a recommendation by a healthcare provider, often resulting in delayed intervention. This phenomenon is particularly prevalent among adolescent orthodontic patients. One study found that approximately 30% of adolescents exhibit delayed treatment behavior, with the primary reasons being fear of the treatment process, financial pressure, lack of awareness about orthodontic treatment, and insufficient parental involvement (5). Treatment delays not only affect the outcomes of orthodontic care but can also increase the complexity and duration of treatment, especially if the optimal treatment window—during the mixed or early permanent dentition stages—is missed (6). Research has shown that patients who miss the ideal treatment period experience significantly reduced outcomes and higher rates of complications during orthodontic care (7).

In addition to the impact on oral health, the relationship between malocclusion and mental health, particularly depressive symptoms, has garnered increasing attention. Adolescents are a high-risk group for mental health issues, and research indicates that approximately 20% of adolescents globally experience some degree of depressive symptoms during puberty (8). Malocclusion may exacerbate psychological issues by affecting the appearance and social interactions of adolescents. One study found that adolescents with severe malocclusion had significantly higher rates of depressive symptoms compared to those with normal dentition (9).

Although some studies have explored the relationship between malocclusion and mental health, little research has delved into the association between delayed orthodontic treatment and depressive symptoms. Given that treatment delays may worsen malocclusion severity and, in turn, negatively impact adolescents’ mental health, it is crucial to further investigate the link between these two factors. This will provide scientific evidence for clinical interventions and public health policy development.

The aim of this study is to evaluate the prevalence of delayed orthodontic treatment among adolescent patients and explore the association between delayed treatment and depressive symptoms. By clarifying the impact of delayed treatment on adolescent mental health, this study holds not only theoretical but also practical significance. Understanding the relationship between delayed orthodontic treatment and depressive symptoms in adolescents can offer new perspectives on addressing mental health issues in orthodontic care and provide dental professionals with a basis for early identification and intervention of depressive symptoms.

## 2 Study participants and methods

### 2.1 Study participants

The participants of this study were adolescent orthodontic patients from Chengde City Oral Hospital, Hebei Province, between May 2023 and April 2024. The inclusion criteria were: (1) adolescents aged between 12 and 18 years; (2) currently undergoing or having previously undergone orthodontic treatment, with a documented delay between the time recommended by the doctor and the actual treatment; and (3) participants and their guardians who provided informed consent to participate in the study. The exclusion criteria were: (1) a history of severe psychiatric disorders (such as major depressive disorder, bipolar disorder, schizophrenia, etc.) or other factors that could affect the assessment of depression; (2) inability to complete the depression assessment due to other health issues; and (3) significant complications during orthodontic treatment that might affect the treatment’s progress and the study’s results. All participants completed the questionnaire in a supervised setting at the clinic. The questionnaires were administered by trained dental nurses and postgraduate medical students who received standardized training before the study. Participants were given verbal instructions, and assistance was provided if they had difficulty understanding any items. Data collection was conducted using paper-based questionnaires, which were then digitized and checked by two independent researchers for accuracy.

### 2.2 Data collection

Data collection included questionnaires, clinical examinations, and patient medical records. Each participant was required to complete the following information:

#### 2.2.1 Questionnaire

**General Demographic Information:** Data were collected on the participants’ age, gender, family economic status, parental education level, marriage status of parents and residence.

**Delayed Orthodontic Treatment Behavior:** Information on the time recommended by the doctor and the actual time of treatment was gathered from medical records and patient self-reports to calculate the delay period. Delayed treatment behavior was defined as a delay of more than 30 days between the recommended and actual treatment time (10).

**depressive symptoms Assessment:** The Beck Depression Inventory for Youth (BDI-Y) (11) was used to assess depressive symptoms. This scale is a widely used and validated tool specifically designed to evaluate depression in adolescents. It consists of 21 items, each scored on a scale from 0 to 3, assessing emotional, cognitive, and behavioral aspects of depression in adolescent. Although validation studies of the BDI-Y in Chinese populations are limited, psychometric research on related versions, such as the BDI-II, has demonstrated good reliability and validity among Chinese samples (12).

#### 2.2.2 Clinical examination

**Treatment type:** According to the treatment plan given by the attending physician at the first visit, including invisible treatment and fixed treatment.

**Malocclusion Severity**: The severity of malocclusion was assessed using standardized tools, such as the Index of Orthodontic Treatment Need (IOTN). The IOTN is an effective instrument for distinguishing different levels of malocclusion severity and provides a basis for analyzing the impact of delayed treatment on oral health (13, 14).

### 2.3 Statistical analysis

The basic characteristics of the study participants (such as age, gender, family economic status, and malocclusion severity) were described using means, standard deviations, and percentages. The distribution of delayed treatment behavior and depressive symptoms was also analyzed. Spearman’s rank correlation coefficient was used to analyze the relationship between delayed treatment time and depressive symptoms scores. Depressive symptoms were treated as a binary variable (e.g., no depressive symptoms vs. depressive symptoms), and a logistic regression model was used to assess the impact of delayed treatment behavior on the risk of developing depressive symptoms.

### 2.4 Sample size and study design

This study utilized a cross-sectional design. The sample size was calculated using the formula: n= *Z*²*p*(1-*p*)/*d*².Where *Z* is the Z-value for the desired confidence level (1.96 for 95% CI), *p* is the expected proportion of delay in orthodontic treatment (estimated to be 0.29 based on previous studies (15)), and *d* is the desired precision (set at 0.05). After calculation, a sample size of 316 participants was required. Considering a 10% non-response rate, the final sample size was determined to be 348.

## 3 Results

### 3.1 Analysis of basic characteristics and depressive symptoms in adolescent orthodontic patients

A total of 450 adolescent orthodontic patients participated in this study, and they were divided into two groups based on the presence of depressive symptoms: the non-depressed group (306 patients) and the depressed group (144 patients). Statistical analysis of the basic characteristics revealed the following significant differences. The mean age in the depressed group was significantly higher than in the non-depressed group (15.88 ± 1.45 years vs. 13.93 ± 1.54 years, *t* = -12.74, *P* < 0.001), indicating that older adolescents are more prone to depressive symptoms. A significant association was found between gender and depressive symptoms (*χ*² = 47.85, *P* < 0.001). Males accounted for 67.32% of the non-depressed group, while females made up 67.36% of the depressed group, suggesting that females are more likely to experience depressive symptoms. There was a significant relationship between parental marital status and depressive symptoms (*χ*² = 6.55, *P* = 0.010). Adolescents from families with marital discord (e.g., divorce or separation) were more likely to experience depressive symptoms (15.28% vs. 7.52%). Family monthly income was significantly associated with depressive symptoms (*χ*² = 16.60, *P* < 0.001). A higher proportion of depressed patients came from low-income (27.33%) and middle-income (35.56%) families. The type of orthodontic treatment was significantly correlated with depressive symptoms (*χ*² = 7.12, *P* = 0.008), suggesting that patients undergoing more complex treatments were more likely to experience depressive symptoms. IOTN was strongly correlated with depressive symptoms (*χ*² = 93.84, *P* < 0.001). Higher IOTN scores, indicating more severe malocclusion, were associated with a higher risk of depressive symptoms. The mean number of days of treatment delay in the depressed group (216.10 ± 170.33 days) was significantly higher than that of the non-depressed group (115.50 ± 131.19 days, t = -6.27, *P* < 0.001), indicating that delayed treatment may play a significant role in the onset of depressive symptoms. See **Table 1** for details.

**Table 1:** Analysis of characteristics and depressive symptoms among adolescent orthodontic patients.

Variables	Total (n = 450)	Non-depressive symptoms (n = 306)	Depressive symptoms (n = 144)	*t/χ²*	*P*
Age, year	14.55 ± 1.76	13.93 ± 1.54	15.88 ± 1.45	-12.74	<.001
Delaytime, days	147.69 ± 152.11	115.50 ± 131.19	216.10 ± 170.33	-6.27	<.001
Gender, n(%)				47.85	<.001
Male	253 (56.22)	206 (67.32)	47 (32.64)		
Female	197 (43.78)	100 (32.68)	97 (67.36)		
Residence, n(%)				2.32	0.128
Urban	236 (52.44)	168 (54.90)	68 (47.22)		
Rural	214 (47.56)	138 (45.10)	76 (52.78)		
Parental education level, n(%)				5.74	0.057
Undergraduate or above	132 (29.33)	83 (27.12)	49 (34.03)		
Undergraduate	122 (27.11)	78 (25.49)	44 (30.56)		
Undergraduate or below	196 (43.56)	145 (47.39)	51 (35.42)		
Marriage status of parents, n(%)				6.55	0.010
Married	405 (90.00)	283 (92.48)	122 (84.72)		
Divorce or other	45 (10.00)	23 (7.52)	22 (15.28)		
Family economic status, n(%)				16.60	<.001
Poor	123 (27.33)	69 (22.55)	54 (37.50)		
Generally	160 (35.56)	106 (34.64)	54 (37.50)		
Good	167 (37.11)	131 (42.81)	36 (25.00)		
Treatment type, n(%)				7.12	0.008
Invisible orthodontic	194 (43.11)	145 (47.39)	49 (34.03)		
Fixed orthodontic	256 (56.89)	161 (52.61)	95 (65.97)		
IOTN, n(%)				93.84	<.001
1	105 (23.33)	91 (29.74)	14 (9.72)		
2	98 (21.78)	86 (28.10)	12 (8.33)		
3	120 (26.67)	83 (27.12)	37 (25.69)		
4	71 (15.78)	24 (7.84)	47 (32.64)		
5	56 (12.44)	22 (7.19)	34 (23.61)		

Based on the total BDI-Y scores, depressive symptoms were further categorized into mild (14-19), moderate (20-28), and severe (≥29) levels. Among the 144 participants identified with depressive symptoms, 57 (39.6%) had mild, 62 (43.1%) had moderate, and 25 (17.4%) had severe symptoms. This distribution supports the interpretation that delayed treatment is not only associated with presence but also severity of depressive symptoms.

### 3.2 Distribution of treatment delay and correlation with depressive symptoms

Among the 450 adolescent orthodontic patients, the mean treatment delay was 147.68 ± 152.11 days. Based on delay duration, participants were categorized into five groups: ≤30 days, 31–90 days, 91–180 days, 181–360 days, and >360 days. The group sizes were 80 (17.78%), 132 (29.33%), 127 (28.22%), 73 (16.22%), and 38 (8.45%), respectively (**Table 2**).

**Table 2:** Distribution of Treatment Delay and correlation with depressive symptoms.

Delay Group (days)		Cases (%)	Item7 Self disgust	Item8 Self criticism	Item13 Social distress	Item14 No sense of value	Depressive symptoms
No delay	≤ 30	80(17.78)	0.55 ± 0.78	0.58 ± 0.78	0.56 ± 0.73	0.43 ± 0.65	10.45 ± 7.83
Delay	31-90	132(29.33)	0.53 ± 0.78	0.57 ± 0.87	0.52 ± 0.79	0.57 ± 0.81	11.75 ± 8.98
	91-180	127(28.22)	0.81 ± 1.09	1.19 ± 1.21	0.60 ± 0.84	0.65 ± 0.87	13.61 ± 10.15
	181-360	73(16.22)	2.25 ± 1.12	1.47 ± 0.96	1.12 ± 1.08	1.30 ± 1.13	22.98 ± 13.33
	> 360	38(8.45)	2.13 ± 1.17	2.63 ± 0.67	2.37 ± 1.00	1.82 ± 0.51	18.45 ± 13.32
*F*			56.59	42.61	40.54	27.76	19.24
*P*			<.001	<.001	<.001	<.001	<.001

**Table 2** also presented the mean scores for total depressive symptoms and four selected BDI-Y items: self-disgust (Item 7), self-criticism (Item 8), social distress (Item 13), and sense of value (Item 14). All variables showed statistically significant differences across delay groups according to one-way ANOVA (all *P* <.001).

The mean total depression score increased progressively across groups, ranging from 10.45 ± 7.83 in the ≤30-day group to 18.45 ± 13.32 in the >360-day group (*F* = 19.24, *P* <.001). Similarly, the mean score for self-disgust ranged from 0.55 ± 0.78 to 2.13 ± 1.17 (*F* = 56.59, *P* <.001), for self-criticism from 0.58 ± 0.78 to 2.63 ± 0.67 (*F* = 42.61, *P* <.001), for social distress from 0.56 ± 0.73 to 2.37 ± 1.00 (*F* = 40.54, *P* <.001), and for worthlessness from 0.43 ± 0.65 to 1.82 ± 0.51 (*F* = 27.76, *P* <.001).

### 3.3 Multivariable logistic regression analysis of treatment delay and depressive symptoms

To further explore the impact of treatment delay on depressive symptoms, a multivariable logistic regression analysis was performed (see **Table 3**). The results showed that in the unadjusted model (Model 1), the longer the treatment delay, the higher the risk of depressive symptoms. In particular, patients with delays of 181–360 days (OR=11.95, 95%CI: 5.50-25.97, *P*<0.001) and those with delays exceeding 360 days (OR=5.73, 95% CI: 2.40-13.68, *P*<0.001) had a significantly increased likelihood of developing depressive symptoms. After adjusting for variables such as age, gender, residence, parental education level, parental marital status, and family monthly income (Model 2), the risk of depressive symptoms remained significantly higher in patients with treatment delays of 181–360 days (OR=4.86, 95%CI: 1.85-12.78, *P*=0.001) and those with delays exceeding 360 days (OR=3.27, 95%CI: 1.16-9.25, *P*=0.026). In the fully adjusted model (Model 3), which included adjustments for treatment type and IOTN score, treatment delays of 181–360 days (OR=4.26, 95% CI: 1.37-13.28, *P*=0.012) and delays exceeding 360 days (OR=3.40, 95%CI: 1.02-11.37, *P*= 0.047) remained significantly associated with depressive symptoms.

**Table 3:** Logistic Regression analysis of delayed medical treatment and depressed mood among adolescent orthodontic patients.

Variables	Model 1 OR (95%CI)	Model 2 OR (95%CI)	Model 3 OR (95%CI)
Delay Group (days)			
≤ 30	1.00 (Reference)	1.00 (Reference)	1.00 (Reference)
31-90	1.33 (0.64 - 2.75)	0.85 (0.36 - 2.02)	0.88 (0.33 - 2.36)
91-180	1.81 (0.89 - 3.70)	1.29 (0.55 - 2.98)	0.93 (0.35 - 2.43)
181-360	11.95 (5.50 - 25.97)	4.86 (1.85 - 12.78)	4.26 (1.37 - 13.28)
> 360	5.73 (2.40 - 13.68)	3.27 (1.16 - 9.25)	3.40 (1.02 - 11.37)

OR, Odds Ratio; CI, Confidence Interval. Statistical significance determined by 95% CI exclusion of 1.

Model 1: Crude

Model 2: Adjust: Age, Gender, Residence, Parental education level, Marriage status of parents, Family economic status.

Model 3: Adjust: Age, Gender, Residence, Parental education level, Marriage status of parents, Family economic status, IOTN.

### 3.4 Sensitivity analysis

To further validate the robustness of the results, we conducted a sensitivity analysis by dividing the delay in treatment into quartiles and performing a multivariable logistic regression analysis (see **Table 4**). The results confirmed that the association between delayed treatment and depressive symptoms in adolescent orthodontic patients remained consistent and robust.

**Table 4:** Sensitivity Analysis.

Variables	Model 1 OR (95%CI)	Model 2 OR (95%CI)	Model 3 OR (95%CI)
Delay time			
Q1	1.00 (Reference)	1.00 (Reference)	1.00 (Reference)
Q2	1.53 (0.78 ~ 3.01)	1.16 (0.52 ~ 2.57)	1.21 (0.49 ~ 3.00)
Q3	1.96 (1.01 ~ 3.81)	1.63 (0.74 ~ 3.59)	1.13 (0.46 ~ 2.76)
Q4	9.05 (4.76 ~ 17.22)	4.59 (2.08 ~ 10.11)	4.10 (1.65 ~ 10.22)

OR, Odds Ratio; CI, Confidence Interval. Statistical significance determined by 95% CI exclusion of 1.

Model1: Crude

Model2: Adjust: Age, Gender, Residence, Parental education level, Marriage status of parents, Family economic status.

Model3: Adjust: Age, Gender, Residence, Parental education level, Marriage status of parents, Family economic status, IOTN.

## 4 Discussion

### 4.1 Analysis of depressive symptoms and demographic factors in adolescent orthodontic patients

This study revealed a high prevalence of depressive symptoms among adolescent orthodontic patients, with 32% of the sample experiencing depressive symptoms, a rate significantly higher than that of the general adolescent population (16). This finding aligns with previous studies that suggest adolescent orthodontic patients face greater psychological pressure compared to their peers due to heightened sensitivity to appearance during the socially sensitive period of adolescence (17). As other studies have indicated, adolescents undergoing orthodontic treatment may experience emotional fluctuations due to factors such as dental malocclusion, changes in facial appearance, and discomfort during treatment (18).

Furthermore, this study found that depressive symptoms were closely related to several demographic factors. Older adolescents were more likely to experience depressive symptoms, possibly due to increased self-awareness and sensitivity to external judgment as they age (19). Gender differences were also significant, with female patients exhibiting a higher rate of depressive symptoms than males, consistent with findings that suggest adolescent females are more susceptible to appearance-related anxiety and social evaluation (20). It is also worth noting that BDI-Y scores are generally higher among females across populations, possibly due to differences in emotional expression and reporting styles. This gender-related scoring tendency should be taken into account when interpreting the observed gender disparity in depressive symptoms.

Family environment was another crucial factor influencing depressive symptoms. This study showed that patients from lower-income families and those with unstable parental marital statuses had significantly higher rates of depressive symptoms. Financial difficulties not only directly affect the timeliness of treatment but also impose additional psychological stress on patients (4). Adolescents from families with marital discord or divorce may lack sufficient emotional support, thereby increasing their psychological burden (21). These findings are consistent with other studies showing that family structure and economic status significantly impact adolescent mental health (22). Although our analysis focused on treatment delay, it is important to note that adolescents may also experience other co-occurring psychosocial or health-related stressors—such as academic pressure, peer conflicts, and undiagnosed anxiety—that independently contribute to depressive symptoms. While participants with severe psychiatric disorders were excluded, these potential confounding influences may partially account for the observed associations and warrant further investigation in future research.

### 4.2 Analysis of treatment delay behavior

This study also revealed that delayed treatment behavior was prevalent among adolescent orthodontic patients, with approximately 82% of patients experiencing varying degrees of delay. Common reasons for delayed treatment included financial difficulties, inadequate understanding of orthodontic treatment, and lack of family support (23). Economically, the study found that adolescents from low-income families were more likely to delay orthodontic treatment, likely due to the significant financial burden posed by the high cost of treatment (5). Additionally, some patients and their families may underestimate the long-term impact of dental malocclusion on oral and mental health, leading to delays in seeking treatment (24).

Lack of family support was also identified as an important factor contributing to treatment delays. Parental involvement in healthcare decisions is crucial, and insufficient parental participation and support may lead to lower compliance with orthodontic treatment, further delaying care (25). Studies have shown that adolescents from divorced families or families with marital discord are more likely to experience treatment delays, which is consistent with the higher incidence of depressive symptoms in these patients (26).

### 4.3 Mechanisms linking treatment delay and depressive symptoms

Further analysis in this study demonstrated a significant association between delayed orthodontic treatment and increased depressive symptoms. Delaying treatment may result in missing the optimal intervention window, leading to the progression of malocclusion and deterioration in oral function (27). These oral health issues can negatively affect adolescents’ appearance and daily functioning, which in turn impact self-esteem and social interactions (28).

Psychological theories suggest that body-related dissatisfaction and perceived social inadequacy are key contributors to emotional distress, particularly during adolescence, a period marked by heightened sensitivity to self-image and peer evaluation (29). In line with this, the present study found significantly higher scores in specific BDI-Y items among patients with longer delays, especially in domains such as self-disgust, self-criticism, social distress, and worthlessness. These item-level differences suggest that treatment delay may intensify negative self-perception and perceived social rejection, both of which contribute to depressive symptoms.

Additionally, prolonged delays may increase patients’ exposure to oral discomfort and treatment-related uncertainty, further contributing to psychological burden (30, 31). Such cumulative stressors may elicit emotional responses such as shame, anxiety, and social withdrawal, ultimately reinforcing depressive tendencies (32). These findings highlight the importance of integrating psychological screening and counseling into orthodontic care for adolescents, particularly those experiencing extended delays. Early identification of negative self-perception and social withdrawal may allow for timely psychological interventions that prevent the escalation of depressive symptoms.

### 4.4 Intervention suggestions to reduce treatment delays and depressive symptoms

To alleviate treatment delays and depressive symptoms among adolescent orthodontic patients, this study proposes several intervention strategies. First, health education is critical. Healthcare providers should enhance educational efforts directed at patients and their families, ensuring that they fully understand the importance of orthodontic treatment and the dangers of long-term treatment delays (33). Additionally, psychological counseling should not be overlooked. For patients exhibiting depressive symptoms, healthcare providers should collaborate with mental health professionals to offer psychological support and assistance.

Financial support is another essential intervention. For patients from low-income families, governments and healthcare institutions should provide more financial aid or insurance coverage to alleviate the economic burden and ensure timely treatment. Finally, effective communication between healthcare providers and patients is crucial during orthodontic treatment. Providers should improve communication with patients and their families, understand their psychological needs, and adjust treatment plans as necessary to reduce psychological pressure and anxiety.

## 5 Study limitations

Although this study revealed a significant association between depressive symptoms and delayed orthodontic treatment, its cross-sectional design prevents the establishment of a causal relationship. Future studies should adopt longitudinal designs to further validate and elucidate the causal link between treatment delay and depressive symptoms. Additionally, the sample in this study was drawn from a specific region, which may limit the representativeness of the findings. Future research should expand the sample size and include participants from a broader range of regions and populations to enhance the external validity of the results. In addition, the BDI-Y was originally developed and standardized in Western populations. Although it has been translated and applied in Chinese adolescents with acceptable reliability, cultural differences in emotional expression and interpretation of items may influence score validity and limit generalizability.

## 6 Future research directions

Future research should focus on longitudinally observing the psychological changes in adolescent orthodontic patients, particularly the long-term effects of treatment delays on depressive symptoms. Additionally, research should explore the effectiveness of various intervention measures, especially multi-dimensional interventions that address financial support, psychological counseling, and health education. These studies will help to develop personalized intervention strategies for adolescent patients, allowing for timely treatment and reduced psychological burden.

## 7 Conclusion

This study demonstrated that the prevalence of depressive symptoms in adolescent orthodontic patients is significantly higher than that in the general adolescent population, and there is a significant association between delayed treatment and depressive symptoms. The study also highlighted the influence of factors such as age, gender, family economic status, and parental marital status on depressive symptoms. Early intervention is crucial for preventing the onset of depressive symptoms. Timely orthodontic treatment not only improves patients’ oral health but also effectively alleviates their psychological stress. Future policy recommendations should focus on increasing attention to adolescent oral and mental health, providing greater financial and social support to ensure comprehensive health protection for adolescent patients.

## Data Availability

The original contributions presented in the study are included in the article/supplementary material. Further inquiries can be directed to the corresponding author.
